# The Difficult Case of Crystallization and Structure Solution for the ParC55 Breakage-Reunion Domain of Topoisomerase IV from *Streptococcus pneumoniae*

**DOI:** 10.1371/journal.pone.0003201

**Published:** 2008-09-12

**Authors:** Maninder K. Sohi, Dennis A. Veselkov, Ivan Laponogov, Xiao-Su Pan, L. Mark Fisher, Mark R. Sanderson

**Affiliations:** 1 Randall Division of Cell and Molecular Biophysics, School of Biomedical and Health Sciences, King's College London, London, United Kingdom; 2 Molecular Genetics Group, Molecular and Metabolic Signalling Centre, Division of Basic Medical Sciences, St. George's, University of London, London, United Kingdom; Massachusetts Institute of Technology, United States of America

## Abstract

**Background:**

*Streptococcus pneumoniae* is the major cause of community-acquired pneumonia and is also associated with bronchitis, meningitis, otitis and sinusitis. The emergence and increasing prevalence of resistance to penicillin and other antibiotics has led to interest in other anti-pneumonococcal drugs such as quinolones that target the enzymes DNA gyrase and topoisomerase IV. During crystallization and in the avenues to finding a method to determine phases for the structure of the ParC55 breakage-reunion domain of topoisomerase IV from *Streptococcus pneumoniae*, obstacles were faced at each stage of the process. These problems included: majority of the crystals being twinned, either non-diffracting or exhibiting a high mosaic spread. The crystals, which were grown under conditions that favoured diffraction, were difficult to flash-freeze without loosing diffraction. The initial structure solution by molecular replacement failed and the approach proved to be unviable due to the complexity of the problem. In the end the successful structure solution required an in-depth data analysis and a very detailed molecular replacement search.

**Methodology/Principal Findings:**

Crystal anti-twinning agents have been tested and two different methods of flash freezing have been compared. The fragility of the crystals did not allow the usual method of transferring the crystals into the heavy atom solution. Consequently, it was necessary to co-crystallize in the presence of the heavy atom compound. The multiple isomorphous replacement approach was unsuccessful because the 7 cysteine mutants which were engineered could not be successfully derivatized. Ultimately, molecular replacement was used to solve the structure by sorting through a large number of solutions in space group P1 using CNS.

**Conclusions/Significance:**

The main objective of this paper is to describe the obstacles which were faced and overcome in order to acquire data sets on such difficult crystals and determine phases for successful structure solution.

## Introduction

It has been shown that topoisomerase lV, a tetrameric complex composed of two ParC and two ParE subunits, is the preferred (or dual) target for many quinolones in *Streptococcus pneumoniae*. DNA topoisomerases play a central role in important cellular processess such as DNA replication, transcription and chromosome segregation [1]–[3]. These enzymes control DNA topology by introducing transient breaks into DNA strands. Type I topoisomerases make a single-stranded DNA break and change the linking number in steps of one, whereas type II topoisomerases catalyse the passage of a DNA duplex through a double-stranded DNA break changing the linking number by two at each catalytic cycle [4]–[6]. Most bacteria possess two type IIA topoisomerases, DNA gyrase and topoisomerase IV [7]–9 which are closely related yet possess distinctive functional activities. The N-terminal domain of ParC (GyrA) catalyses breakage-reunion [10] and its interface with DNA is the primary site of quinolone action. ParE (GyrB) has the ATPase site which is involved in energy transduction. In Gram-negative bacteria such as *E. coli*, it would appear that gyrase is the primary target of quinolone action, whereas in Gram-positive bacteria such as *Streptococcus pneumoniae*, topoisomerase IV or gyrase can be the drug target in a manner dependent on quinolone structure [11], [12]. Interestingly, it has been shown *in vitro* that pneumoccocal topoisomerase IV is more sensitive to quinolone inhibition compared with gyrase, whereas the reverse holds for the *E. coli* enzymes [13]. Also, there appear to be differences in DNA site recognition between pneumococcal topoisomerase IV as compared with *E. coli* gyrase [13]. These aspects have focused attention on both DNA- and quinolone-recognition by the ParC DNA breakage-reunion domain. To gain insight into mechanistic and drug targeting features of topoisomerase IV in Gram-positive bacteria, we sought to express the 55-kDa breakage-reunion domain *of Streptococcus pneumoniae* ParC (ParC55) and to determine its X-ray crystal structure.

Here we go beyond the narrow documentation of final crystallization conditions that produced the refinement-data crystal, to describe the pathway to successful crystallization, data collection and phase determination of this difficult protein with the hope that our experiences will be instructive to others faced with challenging crystallization problems. In this study, the 490-amino acid residue N-terminal DNA breakage-reunion domain of ParC (ParC55), selenomethionine-labelled 1–490 amino acid residue ParC (Se-Met-ParC55) and a variety of cysteine mutants of 1–490 amino acid residue ParC have been expressed in *E. coli* and purified to homogeneity. It proved relatively straightforward to express the pneumococcal ParC55 in *E. coli* and to purify the protein to homogenity. However, problems were encountered at the crystallization stage including twinning and a lack of diffraction for the majority of the crystals grown. Crystallization conditions which included anti-twinning agents, detergents, various precipitants and buffering systems have been tested with an aim to obtain well diffracting single untwinned crystals. Two different methods of flash freezing, one using liquid nitrogen and the other using propane have been compared in order to minimize lattice disordering during freezing. Three different methods of producing heavy atom derivatized crystals, for acquiring data for phasing the structure, have been tested. We obtained two crystal forms, the orthorhombic and the hexagonal. The orthorhombic I222 crystals have unit-cell parameters a = 136.92 Å, b = 137.85 Å, c = 326.02 Å, α = β = γ = 90°. The hexagonal crystal form diffracts to a lower resolution of 5 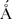 at the synchrotron. Data have been collected to 2.7 Å. As a path to structure solution, several approaches were taken concurrently such as tackling the structure by multiwavelength anomalous diffraction (MAD) employing crystals of selenomethionine-substituted ParC55, using molecular replacement and the GyrA coordinates as a starting structural model, and using multiple isomorphous replacement on the crystals of ParC protein with engineered cysteines, which could potentially be derivatized with a heavy atom through soaking or by co-crystallization [14]–[16]. We have recently reported the full structure solution and refined structure of ParC55, the quinolone target, from Gram-positive bacterium [17]. Carr *et al.* have described the crystallization of two N-terminal fragments of ParC from another Gram-positive bacterium namely *Staphylococcus aureus* [18].

## Results and Discussion

### Purification of ParC55, Se-Met-ParC55 and the mutants of ParC55

**Figure 1:**
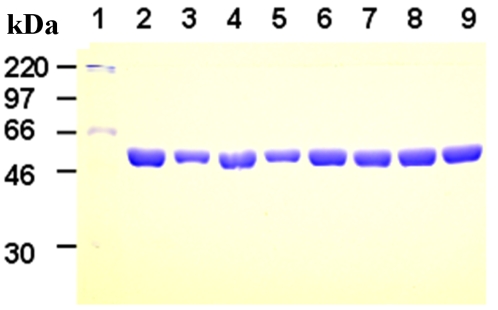
Analysis of purified ParC55 and its cysteine mutants by 10% SDS-polyacrylamide gel electrophoresis. Lane 1, molecular-weight markers; lane 2, wild-type; lane 3, Cys109; Lane 4, Cys110; Lane 5, Cys190; lane 6, Cys294; lane 7, Cys387; lane 8, Cys426; lane 9, Cys437. The molecular weights of the markers are indicated.

ParC55, Se-Met-ParC55 and the mutants of ParC55 bearing hexahistidine tags were expressed at high levels comprising up to 50% to 60% of the total soluble bacterial protein. It was possible to purify the expressed protein to a single band, as detected by SDS-PAGE, by two cycles of chromatography using a nickel-NTA affinity column (Figure 1, lanes 2–9). Storage of the Se-Met-ParC55 at 4°C presented a problem because microcrystals grew in the stock solution at this temperature, which did not fully dissolve and acted as seeds in the crystallization drops, causing growth of a large number of tiny crystals and a substantial precipitation. Therefore, the protein stock was stored at −20°C in the presence of 20% glycerol. Crystals grew reproducibly only from the batches which had not been frozen and thawed more than twice.

### The wild-type ParC55 crystals and data collection

**Figure 2:**
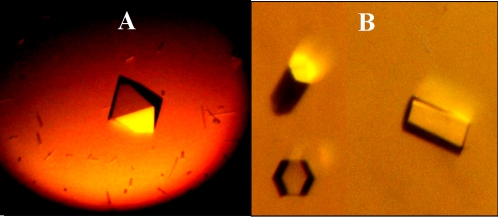
The crystal forms. (A) Orthorhombic crystal of Cys426 grown from 8% PEG 20,000, 200 mM sodium chloride, 100 mM Tris-HCl, pH 5.0, 0.1% sodium azide. (B) Hexagonal crystal of ParC55 grown from 8% PEG 20,000, 200 mM sodium chloride, 100 mM Tris-HCl, pH 7.0, 0.1% sodium azide.

**Figure 3:**
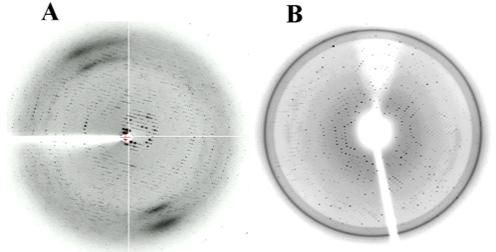
The Diffraction images. (A) Wild type ParC55 twinned crystal grown in a hanging drop containing 6 µl of the protein solution at 3 mg/ml concentration and 6 µl of the reservoir solution containing 200 mM sodium chloride, 0.09 mM sodium azide, 5% (v/v) ethanol, 5% (v/v) isopropanol, 90 mM Tris-HCl, pH 5.5. The diffraction image was taken from the highest resolution (2.7Å) data set collected at ESRF BM 30A. Crystal to detector distance was 340 mm and the exposure time 160 seconds. (B) Mutant Cys426 crystal grown from 6% PEG 400, 1 mM β-mercaptoethanol, 0.05% sodium azide, 200 mM sodium chloride, 100 mM Tris-HCl, pH 6.5. The image was taken from the data set collected at Rigaku Americas Corporation (The Woodlands, Texas, USA) with crystal to detector distance of 336 mm and an exposure time of 5 min.

Two crystal forms of the wild-type ParC55 grew from precipitant solutions containing polyethylene glycol (PEG) 6000, 8000 and 20,000. The crystal form and diffraction quality of the crystals were found to be pH dependent. The capillary mounted, diamond shaped crystals (Figure 2A) which grew at pH between 4.0 and 6.0, diffracted to 3.5 Å whereas the hexagonal plates or needles (Figure 2B), grown at pH above 6.0, diffracted only to 8–5 Å. It was not possible to collect any diffraction data for structure determination using the capillary mounted crystals, because they ceased diffracting after only 1–2 frames. Both crystal forms were difficult to handle during mounting and flash-freezing procedures and had a tendency to crack when the drop containing the crystal was touched even without disturbing the crystal. Only one crystal from each drop could be used given that the remaining crystals started to deteriorate as soon as the well was opened. The inclusion of 25–30% glycerol in the crystallization drop (added to avoid changes in the crystal environment during freezing) was found to be useful in obtaining diffraction to 2.8 Å (Table 1). However, these crystals were slow to grow, requiring 6–8 weeks, and exhibited a high mosaic spread. The best diffracting crystals of the wild-type ParC55 grew from 100 mM Tris-HCl, pH 5.5, 200 mM NaCl, 1 mM â-mercaptoethanol, 0.05% sodium azide and 10% of 1∶1 ethanol-isopropanol as precipitant and diffracted to 2.7 Å (Figure 3A). In this case, the crystallization drops contained 6 ìl protein solution and 6 ìl precipitant solution and the reservoir was 500 ìl. Thus changing the precipitant from PEG to a mixture of ethanol and isopropanol led to obtaining a data set for the first time.

**Table 1:** Crystals tested for diffraction in-house.

Protein	No. of crystals tested	No. of crystals diffracted		
		2.8–4 Å	4.1–5 Å	5.1–14 Å
ParC55 crystals grown from crystallization solution without glycerol	65	3	3	8
ParC55 crystals grown from crystallization solution containing 20% glycerol	29	2	2	0
Se-Met-ParC55 crystals grown from solutions without glycerol	10	4	2	0
Se-Met-ParC55+ethylmercury chloride crystals grown from solutions without glycerol	8	1	0	0
ParC Cys190+ethylmercury chloride crystals grown from solutions without glycerol	6	0	1	0
ParC55 crystals grown from crystallization solution without glycerol and soaked in platinum compounds	65	3	3	8

The cryoprotectant solutions containing 2-methylpropane-1,3-diol (MPD), sucrose or glycerol, which are normally suitable for the other protein crystals grown under the same crystallization conditions as the wild-type ParC55 crystals, did not stabilize these crystals. For example, only one out of 34 crystals tested diffracted to 3.5 Å at SRS Daresbury. The orthorhombic crystals, which grew from a precipitant solution containing a mixture of methanol and ethanol, belong to space group I222 and have unit-cell parameters a = 136.92 Å, b = 137.85 Å, c = 326.02 Å, α = β = γ = 90°. The cryoprotectant that worked best for these crystals was composed of 30% MPD in 150 mM sodium chloride, 0.1% sodium azide, 100 mM Tris-HCl, pH 7.0. Efforts to obtain well diffracting crystals by changing the buffering system from Tris-HCl to citrate, MES or imidazole were not successful. Most of the crystals were either visibly twinned or formed clusters. None of the additives used in our experiments prevented the lattice twinning of these crystals.

### Se-Met-ParC55 crystals and comparison of flash-freezing using liquid nitrogen and liquid propane

Large Se-Met-ParC55 crystals grew from PEG 400 and from PEG 8000 within a few days. The molecular weight of the PEG used for crystallization as well as the method of flash-freezing the crystals proved to be very important for obtaining diffraction data on these crystals. None of the 6 crystals flash-frozen using liquid propane diffracted, whereas some of the crystals frozen in liquid nitrogen diffracted to 2.9 Å. In this respect, the Se-Met-ParC55 crystals contrast with those of the C-terminal domain of UvrB crystals [19] which diffracted to a much better resolution when frozen in liquid propane than in liquid nitrogen. Here we report the comparison of our experiments with these two methods of flash-freezing using two proteins both of which are known to bind DNA. These crystals had the dimensions of 100×100×100 µm and the unit-cell parameters a = 135.19 Å, b = 137.36 Å, c = 323.96 Å, α = β = γ = 90°.

From the analysis of Se-Met-ParC55 MAD data sets it was concluded that the relatively large number of Se sites (16 Se sites per monomer, 4 monomers per asymmetric unit) and twinning of the crystals that increased the number of the Se sites in the asymmetric unit to 128, made the determination and refinement of the Se positions very difficult. Addition of the mercury and the platinum compounds to the crystallization drops led to growth of the hexagonal form, even when the final pH in the drop was kept below 5.5. These crystals either did not diffract at all or diffracted to a very low resolution. Thus, it was not possible to collect any data on the heavy atom derivative crystals beyond 4 Å.

### The mutant ParC55 crystals and diffraction

The cysteine mutants of ParC55 (Cys109, Cys110, Cys190, Cys294, Cys437, Cys387, Cys426) gave the same two crystal forms as the wild-type protein. The hexagonal crystals were very small. The orthorhombic crystals were tested for their diffraction quality and resolution. Both crystal forms were found to be twinned. The number of crystals tested for each mutant and their resolution limits are given in Table 2. The best resolution of 3.25 Å was obtained for the crystals of ParC55 Cys426 without derivatization (Figure 3B). The hexagonal form gave weak diffraction up to ∼8 Å. The mutant Cys426 protein, that gave the best diffracting crystals, was used for derivatization with mercury and platinum compounds both by co-crystallization and by crystal soaking. Only two out of the 49 crystals remained undamaged on soaking in the mercury compounds. The data collected on these crystals exhibited a low occupancy for mercury which was also indicated by fluorescence absorption edge scan. None of the co-crystals with mercury diffracted.

**Table 2:** Mutant ParC55 crystals tested for diffraction in-house.

Mutant	No. of crystals tested	No. of crystals diffracted		
		3.1–4 Å	4.1–6 Å	7–11 Å
Cys109	15	0	0	0
Cys110	7	0	2	5
Cys190	47	5	0	5
Cys294	9	0	0	9
Cys387	10	1	4	5
Cys426	14	1	1	7
Cys426+Hg	49	1	1	0
Cys437	1	0	0	1
Cys190+Hg	49	0	4	43

The reservoir solution conditions, using PEG, for the mutant crystals were similar to those determined for the wild-type ParC55 except that the pH boundary between the hexagonal and orthorhombic crystal forms was higher and the concentration of PEG, required for an optimum crystal size, was lower for the mutant protein than for the wild-type. Unfortunately, the resolution of their diffraction varied from crystal to crystal with two crystals in the same drop behaving differently. From exhaustive crystallization trials, many crystallization conditions were established all of which showed low reproducibility and produced only a very small number of crystals that diffracted. Unlike the wild-type ParC55, the mutant ParC55 crystals did not diffract well and exhibited a high mosaic spread when PEG was replaced with a 1∶1 mixture of ethanol and isopropanol. Moreover, increasing the drop size resulted in just larger crystals which were very similar to the small crystals in terms of diffraction.

### Structure solution for the ParC55

**Figure 4:**
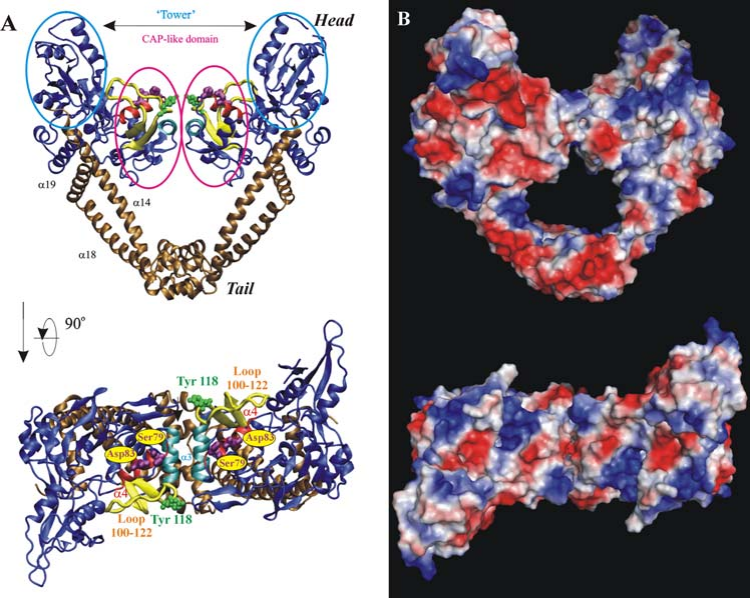
Orthogonal views of the ParC55 biological dimer from *Streptococcus pneumoniae*. (A) Cartoon representation. The ‘towers’ and the CAP-like domains are shown in ice blue; the ‘tails’ along with adjacent helices α14, α18 and α19 are in ochre; the helix α4 in red; the helix α3 in cyan; the 100–122 loop in yellow. The active-site tyrosines are shown in green. Residues responsible for drug-resistance upon mutation are in purple. (B) Electrostatic surface representation. The negatively charged regions are in red and positively charged regions are in blue. Panels were rendered using VMD [27], Pov-Ray and PyMOL [28].

Even though much effort was expended in attempting to solve the Se sites using a wide range of software including the programs SOLVE, SHELXD, and SnB, a clear solution for the sites was not obtained. Although structures for homologous *E. coli* GyrA and *E. coli* ParC were available, our initial attempts to solve the ParC55 structure by molecular replacement using AMoRe [20], [21] failed due to twinning and non-crystallographic symmetry of the crystals as the space group appeared to be I4 but later proved to be I222. A careful analysis of the diffraction data and the results of rotational searches in both space groups helped in establishing the real space group, which was I222 with non-crystallographic symmetry operators mimicking the symmetry of the I4 space group. In addition, the twinning operator (k,-h,l) was equivalent to one of the symmetry operators within I4. Ultimately, molecular replacement was used to solve the structure by sorting through a large number of solutions in space group P1 using CNS [22]. The final structure of the biological dimer of the ParC55 domain of topoisomerase IV from *Streptococcus pneumoniae* is represented in Figure 4. Each monomer contains two distinct regions labeled ‘head’ and ‘tail’. The ‘head’ consists of the DNA binding domain labeled ‘CAP-like’ domain and the ‘tower’. The helices α14, α18, α19 join the head with the tail. More details of the component parts such as α3, α4, α14, α18, α19 helices, active-site tyrosines and 100–122 loops as well as the electrostatic charge distribution have been discussed elsewhere [17].

## Materials and Methods

### Expression plasmid for the 55 kDa N-terminal fragment of ParC (ParC55)

The expression plasmid, encoding the N-terminal 1–490 amino acid residues of *Streptococcus pneumoniae* ParC with molecular weight 55.5 kDa (ParC55), was constructed as described [17]. The expressed ParC55 fragment carried a C-terminal hexahistidine tag and a single amino acid substitution (I489L) resulting from plasmid construction.

### ParC55 expression and purification

*E. coli* BL21(λDE3) pLysS harbouring the expression plasmid was grown in 1 litre LB medium at 30°C to an OD_600_ of 0.6. IPTG was added to 1 mM and the culture was incubated for a further three hours. Cells were harvested by centrifugation and the bacterial pellet was resuspended in 30 ml of binding buffer (20 mM Tris-HCl pH 8.0, 200 mM NaCl, 2 mM β-mercaptoethanol, 10% glycerol). The cell pellet was frozen in liquid nitrogen and stored at −80°C overnight. Cells were thawed on ice and lysozyme was added to a final concentration of 0.02%. After brief sonication to reduce the viscosity, soluble cell extract was recovered by centrifugation and mixed with 1 ml Ni-NTA resin (Qiagen) at 4°C for 2 hours. The protein-bound resin was loaded onto a column, washed with 20 ml of binding buffer and then washed sequentially using the binding buffer containing 20 and 40 mM imidazole. ParC55 protein was eluted with the buffer containing 200 mM imidazole. Fractions were analyzed by SDS-polyacrylamide gel electrophoresis under reducing conditions and the gel was stained with Coomassie blue and those containing the purest protein were pooled together for dialysis against 50 mM Tris-HCl pH 7.5, 200 mM NaCl, 5 mM DTT and 10% glycerol. The protein was further purified by a second cycle of nickel affinity chromatography using the same column.

### Expression and purification of Se-Met-ParC55

A defined medium was used for the production of Se-Met derivative of 1-490 residue fragment of ParC protein. It contained the following components in 1 litre: 6 g Na_2_HPO_4_, 3 g KH_2_PO_4_, 0.5 g NaCl, 1 g NH_4_Cl, 2 ml of 1 M MgSO_4_, 100 µl of 1 M CaCl_2_, 20 ml of 20% glucose, 0.5 ml of 1% thiamine, 10 ml of 400 mg/ml amino acid stock (without methionine), and 1 ml of 50 mg/ml selenomethionine. The expression plasmid for ParC55 was transformed into the host cell B834(λDE3)pLysS. Cells were grown overnight (17 h) in the above medium at 30°C to an OD_600_ of 0.6. IPTG was added to a final concentration of 1 mM and the culture was grown for another two hours before harvesting. Se-Met-ParC55 was purified as described above for ParC55.

### Cys-mutant proteins (Cys109, Cys110, Cys190, Cys294, Cys387, Cys426, Cys437) derived from ParC55

ParC55 (residues 1–490) does not contain any cysteine residues so mutagenesis of the ParC55 expression plasmid allowed the introduction of a single cysteine at position 109, 110, 190, 294, 387, 426 or 437 of the protein. Pairs of 42-mer complementary oligonucleotide primers were designed to regions encompassing the relevant codon which was altered to that for cysteine (TGT or TGC). Mutagenesis was performed with the mutagenic primers and the expression plasmid for ParC55 as a template using the QuikChange site-directed mutagenesis kit (Stratagene) and following the manufacturer's instructions. Plasmids recovered from this procedure were sequenced to confirm that the correct mutation had been introduced. Over-expression was carried out in BL21(λDE3)pLysS and the protein was purified as described for ParC55.

### Screening and optimisation of crystallization conditions

The protein sample to be crystallized was dialysed for 2–4 hours against a solution containing 10% glycerol, 200 mM NaCl, 2 mM β-mercaptoethanol, 20 mM Tris-HCl, pH 7.0. The initial crystallization conditions were screened using PEG ranging from 200 to 20,000 Da., ammonium sulphate, ethanol, methanol, a 1∶1 mixture of ethanol and isopropanol and MPD as precipitants according to the protocol described by McPherson [23]. Three microlitres of the protein solution at 3 mg/ml concentration were mixed with an equal volume of the reservoir solution containing the precipitant in 200 mM NaCl, 2 mM β-mercaptoethanol, 0.1% sodium azide, 100 mM Tris-HCl, pH 3.0–9.5. The drop was equilibrated against 0.5 ml of reservoir solution in a VDX or Linbro plate (Hampton Research Co.). The concentrations of PEG, MPD and saturated ammonium sulphate were 0–30%, 10–80% and 20–80%, respectively. Commercially available sets of solutions including the HR-110 Crystal Screen kit, the HR-112 Crystal Screen II kit, the PEG/LiCl screen, and Grid Screen Polyethylene Glycol 6000 (Hampton Research Co.) were also tested.

### Crystallization trials using additives (anti-twinning agents and detergents)

A number of agents were tested as anti-twinning additives including organic solvents (ethanol, isopropanol, methanol and dioxane), detergents (Tween 20, Tween 80, Triton X-100, Triton X-114, Brij 58, MEGA8 (octanoyl-N-methylglucamide), CHAPS, DDAO (N,N-dimethyldodecylamine N-oxide), CTAB (cetyltrimethylammoniumbromide), divalent and polyvalent ions. Commercially available crystal screen kits were also tested for their effect as anti-twinning solutions by using 10% v/v in the reservoir and the crystallization drop. Compounds containing divalent and polyvalent ions such as magnesium acetate, cadmium acetate, zinc sulphate, zinc chloride, cobaltous(II) chloride, cobaltous sulphate, copper sulphate and nickel nitrate were used at final concentrations between 1 and 4 mM. The final concentration of the detergents used was either 1% or equal to the critical micellar concentration. Additional crystallization experiments were carried out varying the protein concentration from 1.5 mg/ml to 22 mg/ml and sodium chloride from 200 mM to 1 M. Crystallization plates were incubated at 4°C, 22°C and 37°C.

### Heavy atom derivatization of the wild-type ParC55 and ParC55 Cys190 crystals

Co-crystallization experiments were set up in order to derivatize the wild-type ParC55 with 1–3 mM platinum terpyridine, 1–3 mM platinum tetrachloride, 3.9 mM PIP and 1–3.4 mM nickel nitrate. Each compound was dissolved in reservoir solution containing 200 mM sodium chloride, 0.1 mM sodium azide, 5% (v/v) ethanol, 5% (v/v) isopropanol, 100 mM Tris-HCl, pH 5.5 and crystallization was carried out by mixing 6 µl of this solution with an equal volume of the protein solution at 2–3 mg/ml concentration. The volume of the reservoir solution used was 500–550 µl.

Co-crystallization of the ParC55 Cys190 was tested using 100 mM Tris-HCl, pH 6.0–7.0, 200 mM NaCl and 1-3% of PEG 400 as a precipitant in the presence of 0.05–6 mM concentrations of one of the mercury compounds, i.e. p-aminophenylmercury acetate, mercury chloride, mercury acetate, chloromercuryphenyl sulphonic acid and ethylmercury chloride.

### Flash-freezing the crystals and data collection

Crystals were quickly dragged through a drop of the cryoprotectant solution using a loop and flash-frozen either in liquid nitrogen or liquid propane. The ParC55 crystals were screened for diffraction quality using two of our in-house data collection systems, namely a Rigaku R-200 rotating anode generator, Yale mirrors and Rigaku Americas Corporation (The Woodlands, Texas, USA) R-AXIS IIC detector, and an Elliot GX-18 rotating anode generator, Osmic mirrors and Rigaku Americas Corporation (The Woodlands, Texas, USA) R-AXIS IV++ detector. The crystals grown from crystallization solutions containing PEG were cryoprotected using the corresponding crystallization solution containing 25% (v/v) glycerol in place of water. Data were also collected at SRS Daresbury Laboratory stations 7.2, 9.6, 14.2 and 10.1, and ESRF beamlines ID-14.4, ID-29 and BM 30A. For a complete data collection, at least 180° of data with 1° oscillations were collected using an appropriate wavelength with the exposure time set to obtain at least one saturated diffraction spot per frame. Crystal to detector distance varied according to the resolution limits of the crystal. All data collections were conducted keeping the crystal in a nitrogen cryostream.

Prior to data collection, Se absorption edge scans were performed to determine the peak, the inflection point and the remote wavelengths. Data on Se-Met-ParC55 crystals were collected choosing three wavelengths around the Se *K* edge, 0.9793 Å (peak), 0.9795 Å (inflection point) and 0.9392 Å (remote), with an aim to determine the structure using the MAD method. Similarly the data on mercury-derivatized crystals were recorded at 1.00748 Å (peak), 1.00802 Å (inflection point) and 0.98000 Å (remote). The data were indexed, integrated and merged using HKL2000 [24] and XDS [25], [26].
